# Circumcision with plastic Alisclamp technique in 4733 boys: our experiences to reduce complications

**DOI:** 10.3906/sag-2011-199

**Published:** 2021-06-28

**Authors:** Alev SÜZEN, Süleyman Cüneyt KARAKUŞ, Nazile ERTÜRK

**Affiliations:** 1 Department of Pediatric Surgery, Muğla Sıtkı Koçman University, Faculty of Medicine, Muğla Turkey

**Keywords:** Circumcision, Alisclamp, complications, secondary phimosis, bleeding

## Abstract

**Background/aim:**

We aim to report the outcomes of circumcisions performed with Alisclamp and our experiences to reduce the complications.

**Material and methods:**

Complications among circumcised males with Alisclamp between 2015 and 2018 were retrospectively analyzed. Patients were divided into two groups: Group 1 (n = 1429); patients circumcised in 2015–2016 and Group 2 (n = 3304); patients circumcised in 2017–2018. The different technical approaches in Group 2 are as follows: 1) Prevention of bleeding: In Group 2, we didn’t pull the ventral prepuce to reduce the risk of frenulum injury and the foreskin was excised approximately 1–2 mm above the base. 2) Prevention of secondary phimosis: In Group 2, regular manual pressure had been applied to mons pubis and we postponed some of the overweight children’s circumcision. 3) Prevention of excessive foreskin: The clamp was placed carefully to prevent the glans from moving back and forth.

**Results:**

Secondary phimosis was signiﬁcantly lower in Group 2 (p = 0.003). Total bleeding and bleeding requiring suturing were significantly lower in Group 2 (p = 0.001 and p = 0.026, respectively).

**Conclusion:**

Technique-specific complications of Alisclamp can reduce with technique-specific modifications.

## 1. Introduction

Male circumcision involves the surgical removal of the prepuce. It is probably the most commonly performed surgical procedure by pediatric surgeons and urologists worldwide [1,2]. It is performed routinely for religious and social reasons before puberty in Turkey [3,4]. Despite the increased risk of excessive bleeding that can be fatal, hemophiliacs insist on circumcision [5]. Unlike countries, which offer neonatal circumcision due for public health beneﬁts such as prevention of urinary tract infections, penile cancer, and some sexually transmitted infections; in Turkey, it is performed at any age, from newborn period to puberty [2–4]. 

There are myriad methods of circumcision performed worldwide. In general, these methods can be classified into two main groups; (1) conventional surgery techniques and (2) multiple device-assisted techniques with each technique havings its own advantages, limitations, and complications. Alisclamp is the one of the devices designed to assist circumcision. Due to its shorter duration of procedure, ease of application, reduced complications, and better cosmetic appearance compared to conventional surgical circumcision, this minimally invasive technique has been suggested as the circumcision procedure of choice [3,4]. In Turkey approximately 99% of the male population undergoes circumcision and the number of circumcisions performed in hospitals is very high [3,4]. Since 2015, we have started to perform Alisclamp-assisted circumcision due to its short duration. With this study, we aim to report the outcomes of our circumcisions performed with a plastic-Alisclamp device and our experiences to reduce the complications.

## 2. Materials and methods

Data for 4733 males circumcised with Alisclamp in Muğla Sıtkı Koçman University Research and Training Hospital between 2015 and 2018 were retrospectively analyzed. Patients were divided into two groups according to our experience period: Group 1 (n = 1429) and Group 2 (n = 3304) consists of patients circumcised with Alisclamp in 2015–2016 and 2017-2018, respectively. 

Children with genital anomalies or history of bleeding disorders were excluded from the study. All of the circumcised males were followed up 6 months for the possible complications such as bleeding, secondary phymosis, excessive foreskin, and infection. Circumcisions were performed by 3 pediatric surgeons from the same institution. 

### 2.1. Technique 

**Figure 1:**
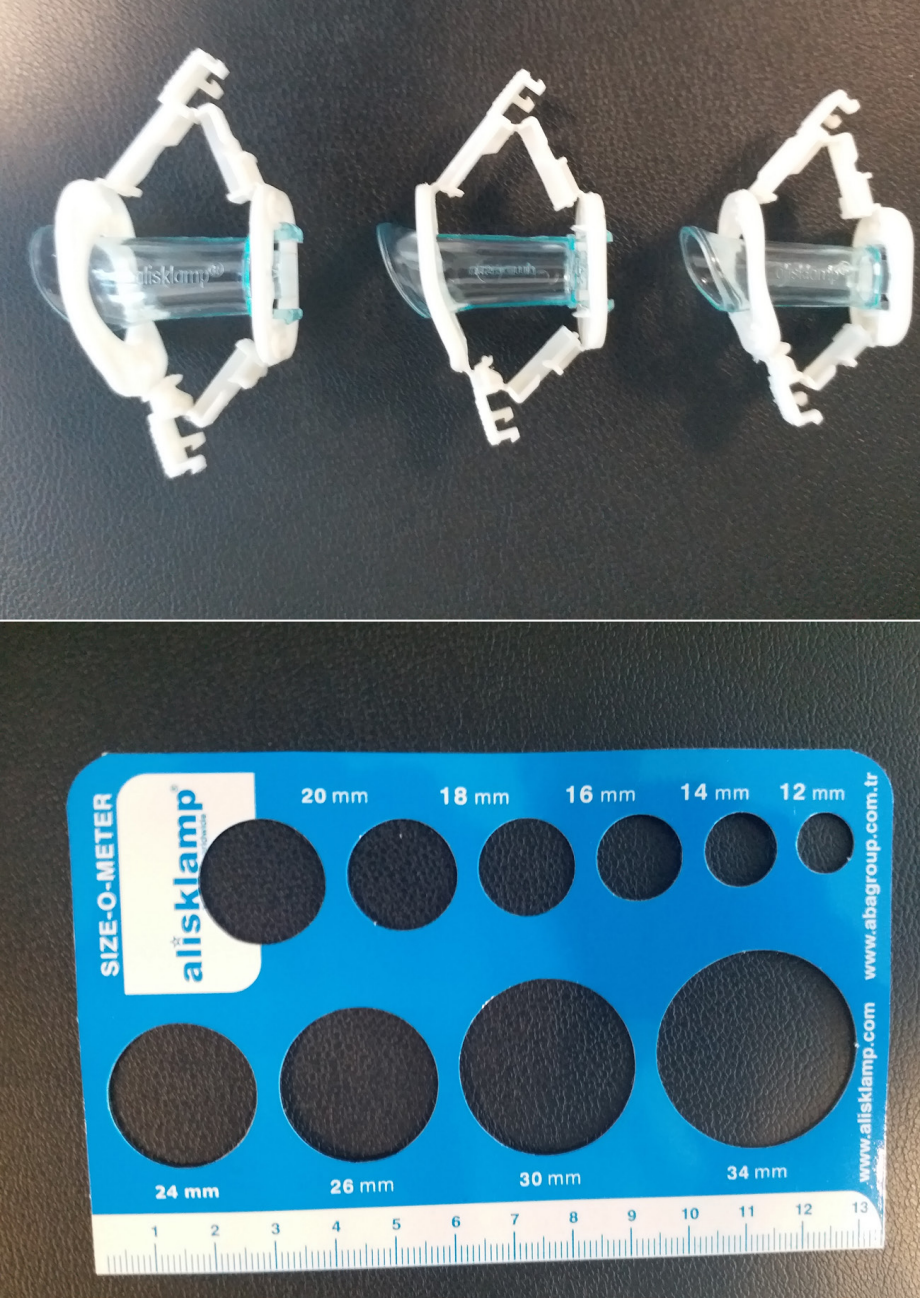
Alisclamp and a special measuring tape with 10 circular holes to determine the appropriate clamp size that just encircled the glans penis at the level of corona

After standard sterile cleaning and draping of the operation site, dorsal penile nerve block was applied with the mixture of 1–5 mg/kg lidocaine HCl 1% and 1–3 mg/kg Bupivacaine HCl. The procedure began approximately 15 min following the application of the local anaesthesia. Alisclamp consists of separate inner tube and an outer ring with the locking arms at the side. It is available in various sizes to accommodate all ages, from newborn to tenageers. Circumcision kit has a special measuring tape with 10 circular holes to determine the appropriate clamp size that just encircled the glans penis at the level of corona (Figure 1). Size 10–14mm clamps are usually preferred for the children up to 2 years of age, and size 14–20 mm clamps for older children. Initially, the prepitium was retracted completely. When the normal glans was exposed, the inner tube of Alisclamp was placed over the glans penis and the retracted foreskin was pulled over this tube. If the prepitial orifice is too narrow for the replacement of retracted foreskin, a dorsal slit was performed. Then, the outer ring was placed over the foreskin. Once the enough foreskin pulled and the urethral meatus in the natural position observed, the clamp was locked. The foreskin distal to the outer ring was excised circumferentially with a surgical blade. Wound care and dressing was not necessery after the procedure. The clamp was removed between 48–96 h depending on the age of the child. St. Jhon’s wort oil (Hipericum perforatum) was applied 2 h before the clamp removal. This application aids in detaching the adhesion between the clamp and the cut wound edges. The patients were regularly followed after the clamp removal at 1 week, 2 months, and 6 months postoperatively, and longer as required.

### 2.2. Modifications 

As our experience has increased, we have implemented following modifications since January 2017 to reduce the risk of bleeding, secondary phimosis, and excessive foreskin.

#### 2.2.1. Prevention of bleeding

**Figure 2:**
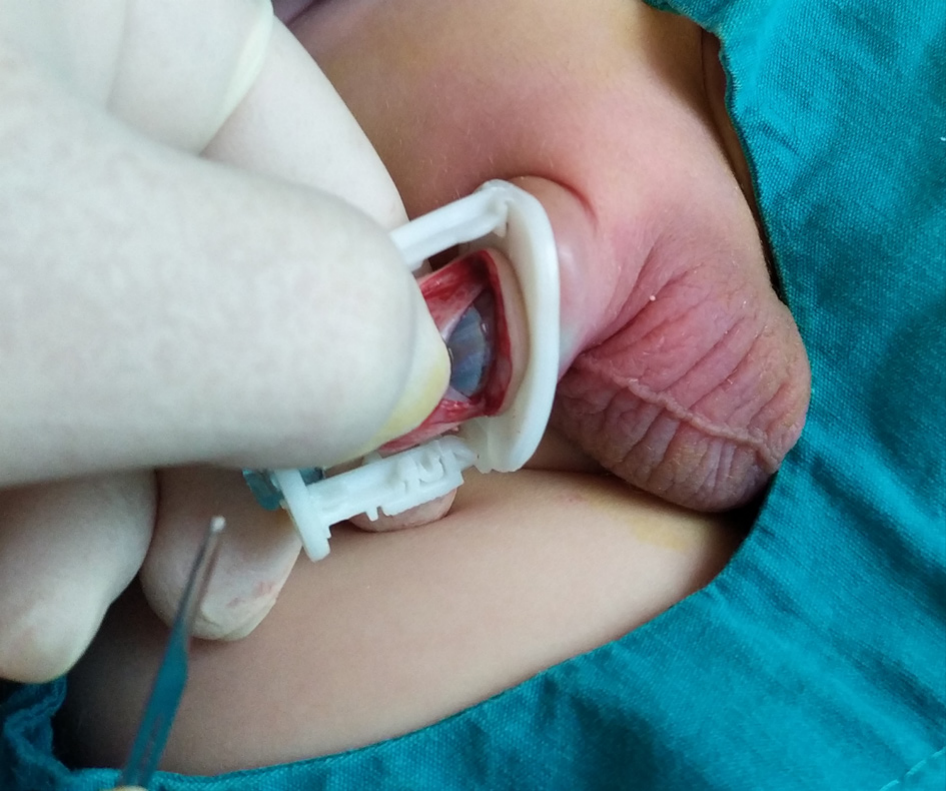
Excision of the foreskin approximately 1–2 mm above the base in Group 2.

a. We noticed that most of the bleeding arised from frenulum. Therefore, we dissected prepuce from the epithelium of glans more gently at the side of frenulum. If we still detected bleeding from the frenulum, we used a bipolar cautery before the clamp, which was placed over the glans in Group 2. 

b. After we placed outer ring of clamp, we didn’t pull the ventral prepuce to reduce the risk of frenulum injury in Group 2. 

c. Spontaneous premature removal of clamp was another cause of hemorrhage. We paid more attention to the clamp size to reduce the risk of cut edges detachment from the clamp. Furthermore, the foreskin was excised from the base of the ring in Group 1, whereas approximately 1–2 mm above the base in Group 2 (Figure 2).

#### 2.2.2. Prevention of secondary phimosis 

**Figure 3:**
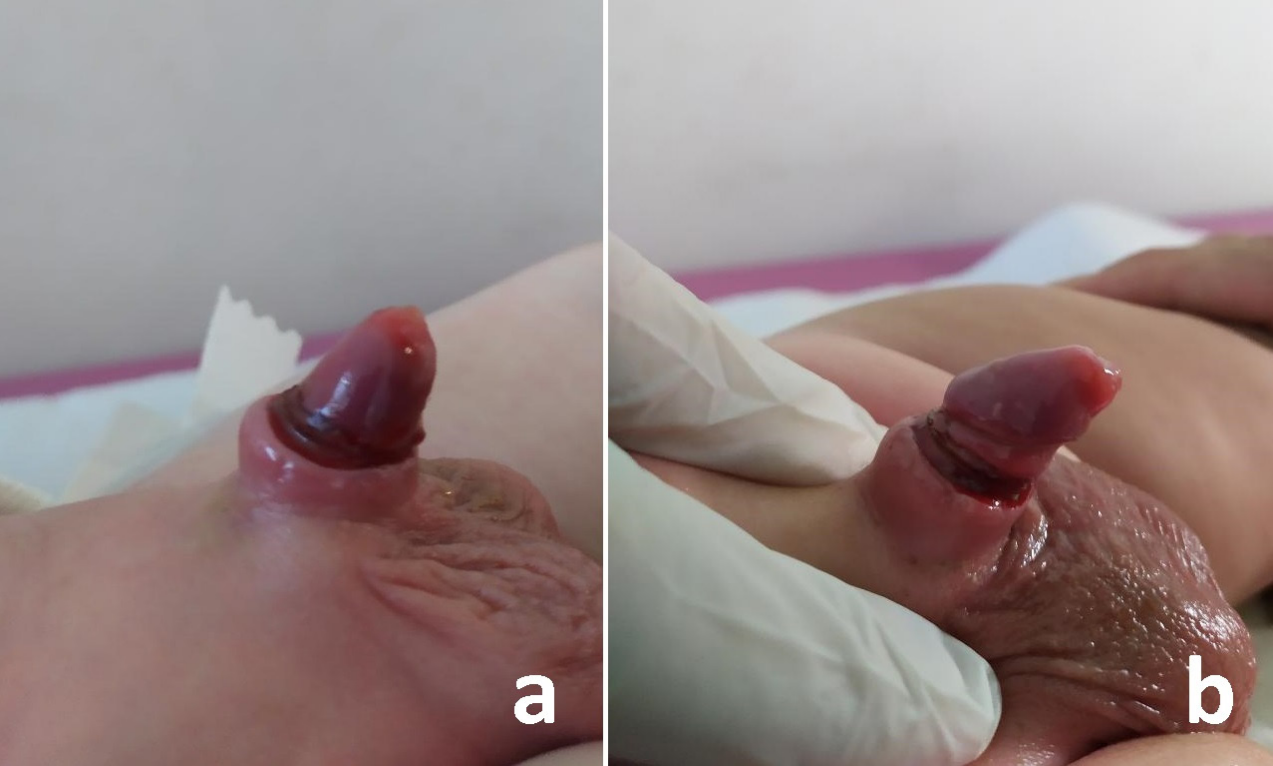
of regular manual pressure to mons pubis (regular retraction) in children with excessive pubic adipose tissue.

a. Application of regular manual pressure to mons pubis (regular retraction) was teached to the family in Group 2 in order to retraction of the penil skin for 2–3 months (Figure 3). This is especially important for children with excessive pubic adipose tissue that leads to inward folding of insicion line and healing with stricture. 

b. We detected that all children in Group 1 with secondary phimosis were >95 percentile on body mass and had excessive pubic adipose tissue. After January 2017, we mentioned the risk of secondary phimosis to the parents of overweight children with excessive pubic adipose tissue. Then, some of them decided to postpone the circumcision. 

#### 2.2.3. Prevention of excessive foreskin 

**Figure 4:**
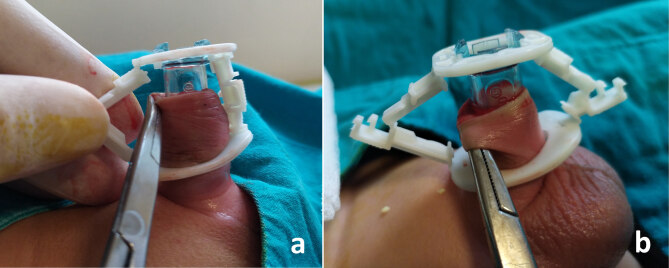
Pulling out the mucosa with a mosquito clamp when folding is detected.

Sometimes the mucosa can be folded under the inner tube and remain longer than we estimated. Therefore, clamp was placed carefully to prevent the glans from moving back and forth. We also pulled out mucosa with a mosquito clamp if there was detected folding (Figure 4).

### 2.3. Statistics

Data collected included age, complications, and necessity of secondary surgery procedures in the presence of complications. The statistical analyses were performed using the SPSS Statistics v: 20.0 for Windows (IBM SPSS Inc., Chicago, IL, USA). The data was expressed as the mean ± standard deviation. The Student’s t test is used to compare the means of age. The Pearson’s chi-square test or Fisher’s exact test, when one or more of the cells have expected frequency of less than 5, were used to compare complications of circumcision between two groups for statistical analysis. p < 0.05 was accepted as statistically significant.

## 3. Results

A total number of 4733 children were circumcised utilizing Alisclamp; 1429 in Group 1 and 3304 in Group 2. The mean age was 36.15 ± 36.90 months in Group 1 and 35.88 ± 128.75 months in Group 2. There was no significant difference between the groups in terms of age (p = 0.938). Complications with Alisclamp technique and the necessity of secondary surgical procedure in the presence of complications are shown in Table.

**Table:** Complications of circumcision with Alisclamp.

Complications	Group 1 (n = 1429)	Group 2 (n = 3304)	p
Secondary phimosis	14 (0.98%)	10 (0.3%)	p = 0.003*
Requirement of reconstructive surgery in secondary phimosis	2 (0.14%)	1 (0.03%)	p = 0.218**
Bleeding	14 (0.98%)	8 (0.24%)	p = 0.001*
Requirement of suturing in bleeding	6 (0.42%)	3 (0.09%)	p = 0.026**
Excessive foreskin	7 (0.49%)	2 (0.06%)	p = 0.004**

**Figure 5:**
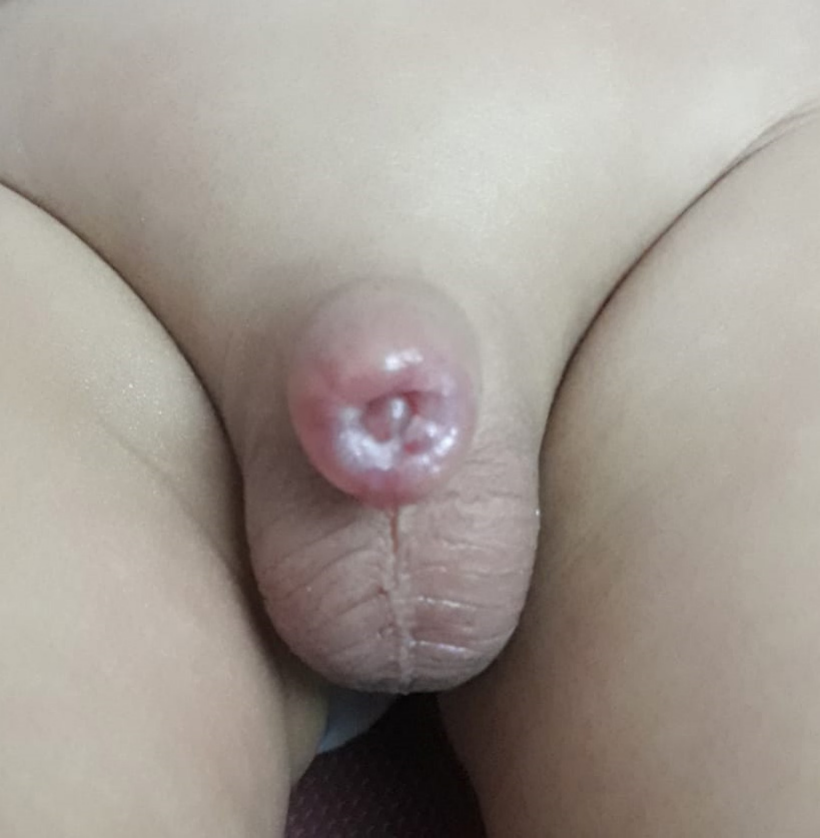
Constrictive ring above the glans penis created by healing of wound edges with stricture, named as secondary phimosis.

The most common complication encountered after cirumcision with Alisclamp was secondary phimosis, which is a constrictive ring above the glans penis created by healing of wound edges with stricture (Figure 5). Secondary phimosis was determined in 14 patients in Group 1. Twelve of them were improved by conservative therapy, consisting of application of regular manual pressure to mons pubis in order to retraction of the penil skin. Secondary phimosis was determined in 10 patients in Group 2. Only 1 patient required reconstructive surgery, the others were improved by ongoing conservative therapy for 3 months. Secondary phimosis was signiﬁcantly lower in Group 2 (p = 0.003). Requirement of reconstructive surgery for secondary phimosis was lower in Group 2, but it was not statistically significant ( p = 0.218). Interestingly, all children in Group 1 and 2 with secondary phimosis were <2 years age and > 95 percentile on body mass.

Bleeding was the second most common complication, but in most instances, it was controlled by applying compressive dressing. Total bleeding and bleeding requiring suturing were significantly lower in Group 2 (p = 0.001 and p = 0.026, respectively). 

Although excessive foreskin was detected in 7 patients in Group 1 and 2 patients in Group 2, and the difference between the groups was statistically significant (p = 0.004). None were cosmetically long enough to require an early operation, so we decided to wait until puberty for all of them. 

Total number of complications encountered after Alisclamp technique was 35 (2.45%) in Group 1 and 20 (0.6%) in Group 2 (p < 0.001).

## 4. Discussion

Circumcision is one of the oldest and most commonly performed surgical procedures in the world [6]. There are multifarious acceptable circumcision techniques described. Before 2016, we preferred to perform circumcision by conventional techniques. With the increase in the number of circumcision requests, we have started to the Alisclamp technique which has been demonstrated to be a more safe, easy, and quick method [3,4]. It is also known as Smart Clamp Circumcision Device in the literature [7]. There is no need for suturing and haemostasis that shortens the operation time. Additionally, it can easily be performed without assistance and excessive tools. Only one or two mosquitos are enough to pull the mucosa and foreskin over the clamp. As our experience has increased, we decided to implement several changes on January 2017 to reduce the rates of preventable complications. 

Secondary phimosis was the most common complication (0.5%) encountered after Alisclamp circumcision in our study. Senel et al. used the term “buried penis” instead of secondary phimosis in the literature [3,4]. We think that they used this term as the penis was embedded in pubic adipose tissue. There is indeed a constrictive ring above the glans penis created by healing of wound edges with stricture, and as in pathologic phimosis, the foreskin of the penis cannot be pulled back past the glans secondary to this scarring. Therefore, secondary phimosis and also acquired phimosis, iatrogenic phimosis or trapped penis are better terms, rather than buried penis [8,9]. It is stated that excessive removal of the shaft skin, slippage of the inner layer of the prepuce and a prominent suprapubic fat pad are the causes of this complication [9,10]. Similar to our results, Senel et al. reported that the children below 2 years age, and over 95 percentile on body mass were under the risk of secondary phimosis. We suggested that the high rate (1.04 %) of secondary phimosis reported in the literature can be reduced by expressing this complication to the parents of children at risk or application of regular manual pressure to pubic adipose tissue [4]. Kidger et al. also recommended regular retraction of the outer layer immediately to the children at risk of acquired phimosis. On the other hand, they stated that corrective surgery is invariably required for the children with post-circumcision cicatrix and it should be performed at least 3 months later to reduce bleeding due to inflammation [8]. Alternatively, we prefered to continue with regular retraction and only patients with persisted secondary phimosis for more than 1 year were re-circumcised. In total, of the patients with secondary phimosis, 21 (87.5%) patients improved without re-operation. In addition to regular retraction, we considered that spontenous resolution of scarring in time may also contribute to healing.

Bleeding is reported as the most common complication of conventional circumcision with a rate up to 19.7% [4,10,11]. On the other hand, it was the second most common complication in our study. We noticed that the most common cause of bleeding was torn frenular artery in Group 1, which can be identified easily by the presence of rapid bleeding rather than minor ooze and the fact that it does not usually stop spontaneously. Bleeding from frenulum was detected both just after the circumcision and also hours later. We performed gentle disection of frenulum and, if necessary, used bipolar cautery in Group 2 in order to reduce early bleedings. Late bleedings in Group 1 were attributed to the press or traction to the Alisclamp done by patients and their diaper or underwear. Therefore, following the placement of outer ring, we didn’t pull the ventral preputium to reduce the risk of frenulum injury in Group 2. Premature removal of clamp due to slippage of cut edges was the another cause of late bleeding in Group 1. We paid more attention to the clamp size and excised the preputium approximately 1–2 mm above the base of ring in Group 2. Additonally, Senel et al. reported that bleeding after circumcision with Alisclamp usually occured 24 h after the remowal of clamp due to unexpected detachment of the crust before the completion of appropriate healing. They suggest disposable foam or special underwear with a concave plastic protection on the front side to reduce the risk of frictional trauma applied to the wound. Since we routinely recommend to parents the protection of wound with special underwear, we did not encounter such a bleeding.

Excessive foreskin was the third complication observed among the children circumcised with Alisclamp. We specified that inadvertently folded mucosa was the cause of excessive foreskin in Group 1. Karadag et al. prospectively analyzed outcomes of the SmartClamp circumcision and the classic surgical dissection technique. They reported shorter operation time, similar cosmetic results and complication rates, but also longer mucosal length in patients circumcised with SmartClamp [12]. The modifications including the placement of Alisclamp carefully to prevent the movement of glans penis, and also pulling out the mucosa with a mosquito clamp, if there was detected folding, were statistically reduce the risk of excessive foreskin in our study.

**Figure 6:**
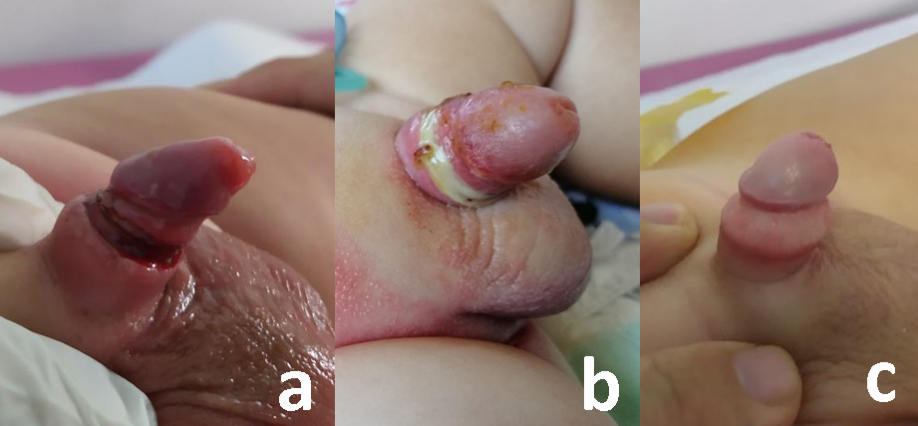
The darkish necrotic wound tissue seen after the removal of clamp, b. yellowish-whitish appearence of necrotic wound tissue a few days later and, c. good cosmetic appearance of penis circumcised with Alisclamp.

The infection rates of plastic clamp circumcision techniques were reported lower or similar compared to conventional circumcision in the literature [4,12]. Interestingly, we did not encounter any infection. Most surgeons defined the infection as increased hyperaemia and oedema with pus. On the other hand, statistically more common penile oedema was reported with Smartclamp as our observation [12]. Furthermore, hyperaemia and oedema of these patients improves within a longer time. The darkish necrotic wound tissue seen after the removal of clamp becomes yellowish-whitish within a few days (Figure 6a, 6b, and 6c). Most health care providers misdiagnose it as an inflammation. We did not administer antibiotics to the patients with hyperemia, oedema and yellowish-whitish tissue, We did not detect any of the reported rare complications such as chordee, meatal stenosis, urethracutaneous fistula, glanular necrosis, and glanular amputation. 

## 4. Conclusion

Circumcision with alisclamp is a safe and easy method with shorter operation time, better cosmetic apperance and lower complications. Technique-specific complications of Alisclamp can be reduced with technique-specific modifications.

## Informed consent

This study was approved by the local ethics committee of the Muğla Sıtkı Koçman University Faculty of Medicine (02.07.2019∕115) and performed in accordance with the Helsinki Declaration of the World Medical Association.
